# A vacuum-sealed miniature X-ray tube based on carbon nanotube field emitters

**DOI:** 10.1186/1556-276X-7-258

**Published:** 2012-05-17

**Authors:** Sung Hwan Heo, Hyun Jin Kim, Jun Mok Ha, Sung Oh Cho

**Affiliations:** 1 Department of Nuclear and Quantum Engineering, Korea Advanced Institute of Science and Technology, Daejeon, 305-701, Republic of Korea; 2 Particla Co., Ltd, Daejeon, 305-701, Republic of Korea

## Abstract

A vacuum-sealed miniature X-ray tube based on a carbon nanotube field-emission electron source has been demonstrated. The diameter of the X-ray tube is 10 mm; the total length of the tube is 50 mm, and no external vacuum pump is required for the operation. The maximum tube voltage reaches up to 70 kV, and the X-ray tube generates intense X-rays with the air kerma strength of 108 Gy·cm^2^ min^−1^. In addition, X-rays produced from the miniature X-ray tube have a comparatively uniform spatial dose distribution.

## Background

A miniature X-ray tube is a small X-ray generation device generally with a diameter of less than 10 mm [1-5]. Because of the feasible installation in a spatially constrained area and the possibility of electrical on/off control, miniature X-ray tubes can be widely used for nondestructive X-ray radiography, handheld X-ray spectrometers [1,2], electric brachytherapy, and interstitial or intracavitary radiation therapy or imaging with the substitution of radioactive isotopes [3-5]. Miniature X-ray tubes have been developed mostly using thermionic electron sources [3,4] or secondary X-ray emission [5].

Meanwhile, X-ray tubes based on carbon nanotube (CNT) field-emission electron sources have been extensively developed because CNT emitters have several advantages compared with thermionic electron sources. The advantages of CNT emitters include (1) cold electron sources, and hence, little heat is generated inside the tube [6] which is important for the minimization of an X-ray tube; (2) simplicity and easy controllability in a pulse operation [7,8]; (3) high current density for electron and X-ray microscopy devices [9,10]. Several types of X-ray tubes have also been developed using CNT field emitters [11-15]. However, the miniature X-ray tubes are mostly not vacuum-sealed and thus should be operated in a vacuum chamber or with a vacuum pump. In addition, the maximum operating voltages of the miniature X-ray tubes were less than 30 kV. As a consequence, the X-ray tubes have limited practical applications.

In this paper, we report that we have developed a vacuum-sealed miniature X-ray tube using a CNT field emitter. The miniature X-ray tube can be operated up to 70 kV and produces X-rays with very high intensities and a comparatively uniform spatial distribution.

## Methods

### Fabrication of the miniature X-ray tube

**Figure 1:**
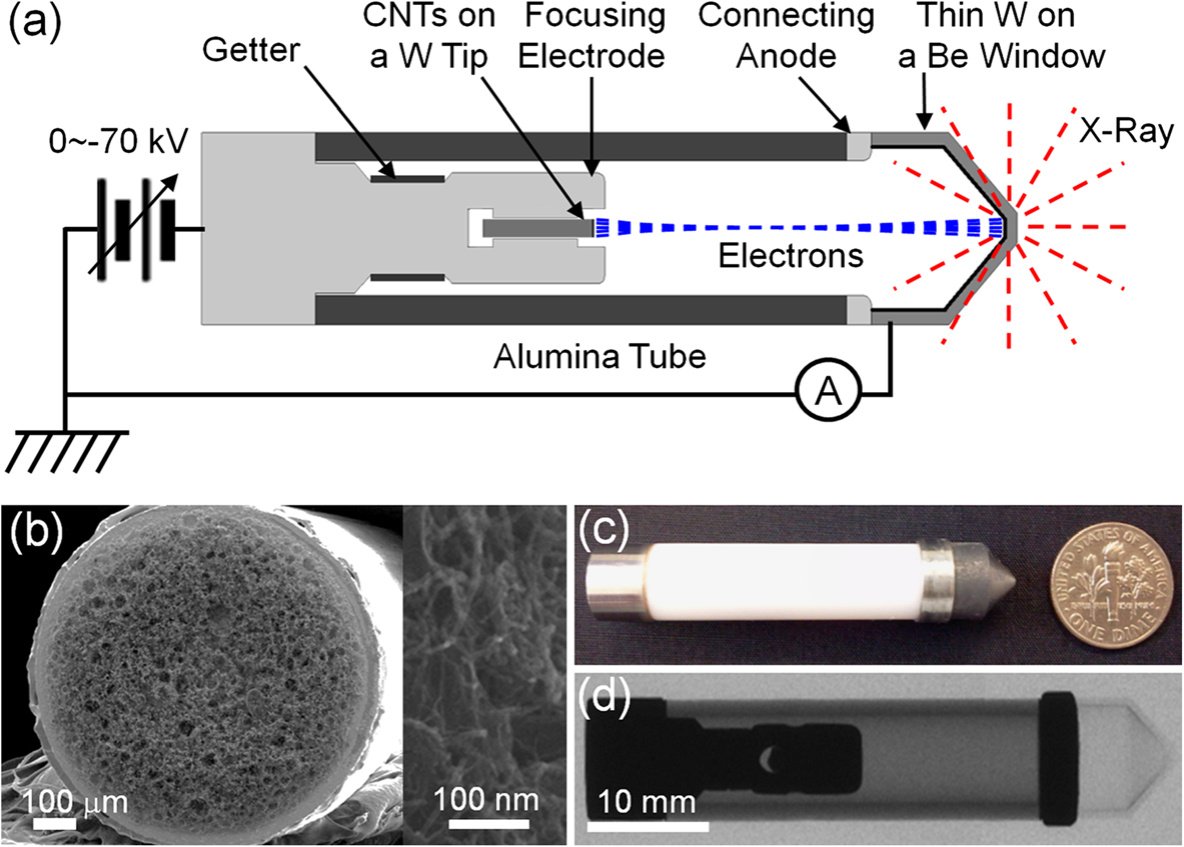
**Schematic diagram, SEM image, photograph, and X-ray radiograph. **(**a**) Schematic diagram of the vacuum-sealed miniature X-ray tube. (**b**) SEM image of the CNT cathode. (**c**) Photograph and (**d**) X-ray radiograph (XR) of the fabricated miniature X-ray tube. The XR has been taken by a microfocus X-ray imaging system (Nikon Metrology XTV160, Nikon Co., Shinjuku, Tokyo, Japan).

The schematic diagram of the miniature X-ray tube is shown in Figure 1a. The X-ray tube has a diode structure, which consists of a CNT cathode tip and a focusing electrode on one side and a conical-shaped transmission-type X-ray target on the other side. An alumina ceramic tube (inner diameter 7 mm, outer diameter 10 mm) is used for the high-voltage insulation between the cathode and the X-ray target. The CNT cathode was fabricated by sintering a CNT paste mixture comprising single-walled CNTs (CNT SP95, Carbon Nanomaterial Technology Co., Ltd., Gyeongbuk, South Korea) and silver nanoparticles on a flat ended tungsten (W) wire with the diameter of 0.8 mm [16]. The scanning electron microscopy (SEM) images show that CNTs were uniformly coated on the W surface (Figure 1b). The CNT cathode tip is installed inside the focusing electrode. The shape of the focusing electrode was determined by the EGN2 code (GA and WB Hermannsfeldt, Los Altos, CA, USA) [17] to focus an electron beam generated at the cathode tip and accordingly to make the beam reach the X-ray target without hitting the ceramic tube. Small loss of the beam at the ceramic tube can induce high-voltage breakdown, and thus, the electron beam optics should be carefully designed not to induce a beam loss in the miniature X-ray tube. The position of the cathode inside the focusing electrode affects both the emission current and the electron beam trajectory, and it was determined by the EGN2 code and by experiment. A conical-shaped transmission type was employed for the X-ray target. The geometry of the X-ray target was determined using the Monte Carlo simulation to produce X-rays with a better spatial uniformity [18]. The X-ray target was fabricated by coating W on a conically machined beryllium (Be) X-ray window using a magnetron sputter. The thickness of the coated W film is 1.5 μm, which is optimized to produce a maximum X-ray output for a given electron beam input [19].

Figure 1c,d show a photograph and an X-ray radiograph (XR) of the fabricated miniature X-ray tube, which show the exterior and the interior of the tube, respectively. The diameter of the X-ray tube is 10 mm, and the total length is 50 mm. The weight of the tube is only 14.5 g. All of the connection parts of the X-ray tube are tightly vacuum-sealed. The both ends of the alumina ceramic tube were vacuum-brazed with a focusing electrode assembly and a connecting anode, respectively. Both electrodes were made of Kovar (Carpenter Technology Corporation, Reading, PA, USA) that has a similar thermal expansion coefficient to alumina. The connecting anode was used to interconnect a ceramic tube and a Be X-ray window that have different thermal expansion coefficients. The connecting anode and the Be window were also vacuum-brazed. All the components of the X-ray tube were baked at 550°C for 10 h, and subsequently, these were brazed through a single-step brazing process at 680°C for 30 min in a vacuum furnace. Before the brazing process, electron emission and transport tests of the X-ray tube have been carried out inside a vacuum chamber. The position of the cathode inside the focusing electrode could be finely controlled through this process. A non-evaporable getter film was installed around the focusing electrode to evacuate the X-ray tube. The getter was activated during the brazing process. The outer part of the sealed X-ray tube, except the target, was covered with a layer of silicone resin to improve high-voltage insulation between the cathode and the X-ray target. We observed that the fabricated X-ray tube was stably operated up to 70 kV without any high-voltage breakdown or discharge at both the inner vacuum side and the outer air side.

## Results and discussion

### Performance and characterization of the X-ray tube

**Figure 2:**
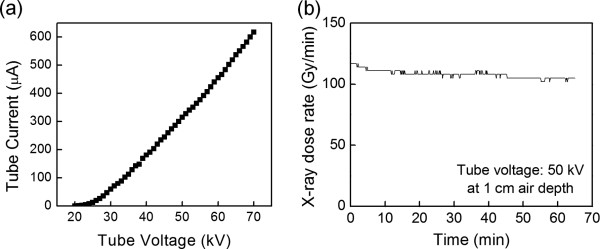
**Current–voltage characteristics, X-ray dose rate, and stability of miniature X-ray tube. **(**a**) Current–voltage characteristics of the fabricated miniature X-ray tube. (**b**) The X-ray dose rate and stability of the miniature X-ray tube.

Figure 2a shows the typical current–voltage (*I*-*V*) characteristics of the fabricated miniature X-ray tube. The current corresponds to the electron beam current arriving at the X-ray target, and the voltage is the tube voltage that is applied between the cathode and the X-ray target. Hence, the *I*-*V* plot represents the operating characteristics of the vacuum-sealed miniature X-ray tube with a diode structure. It should be noted that there was no observable difference between the emission current measured at the CNT cathode and the current reaching the X-ray target. This reflects that an electron beam generated at the CNT emitter has arrived at the X-ray target without loss. The X-ray tube was operated in a negative cathode-bias mode: the cathode and the focusing electrode were floated in negatively high voltage while the X-ray target was grounded. The turn-on voltage at 10 mA/cm^2^ was 29 kV. When the tube voltage was increased to 70 kV, the current at the X-ray target came to 617 μA, corresponding to 0.123 A/cm^2^. The current fluctuation was measured to be *ca*. ±2% at 50 kV; the X-ray tube could not be operated for a long time due to a heat accumulation at the target at the voltage higher than 60 kV. In our X-ray tube, the CNT cathode was inserted in the focusing electrode, and thus, the insertion position of the cathode should be carefully determined because the relative position affects the turn-on voltage and the emission current. In Figure 2, the CNT cathode was inserted 0.5 mm inside the focusing electrode. Prior to the brazing, we investigated the effect of the insertion position on the *I*-*V* characteristics. The turn-on voltage corresponding to 10 mA/cm^2^ was dramatically increased to 8, 19, 29, and 40 kV, and accordingly, the emission current decreased when the insertion position was changed to 0, 0.3, 0.5, and 0.7 mm, respectively. In a diode geometry, current cannot be adjusted at a fixed voltage unlike a triode geometry that has a grid. However, by changing the insertion position of the cathode, a various current selection is available in our X-ray tube.

Figure 2b shows the dose rate of X-ray that is produced from the miniature X-ray tube and the stability of the dose rate with time. The dose rate was measured with an ionization chamber and an electrometer (PTW 34013 and Unidos-E both from PTW, Freiburg, Germany) at 1 cm apart from the X-ray tube in air. The air kerma strength of the X-ray tube operating at 50 kV with the tube current of 252 μA was as high as 108.1 Gy·cm^2^ min^−1^, which is approximately 15 times higher than that of a 10-Ci HDR ^192^Ir radioisotope source [20] that is widely used for brachytherapy. The fluctuation of the X-ray dose rate was as low as ±2.7%. In addition, the X-ray tube has worked for over 2 months with no significant change in the electron beam current (0.25 mA at 50 kV) and the X-ray dose rate. The X-ray tube has been continuously operated for approximately 1 h/day. Consequently, the developed miniature X-ray tube produces high enough X-ray output and exhibits very good short-term and long-term stabilities.

**Figure 3:**
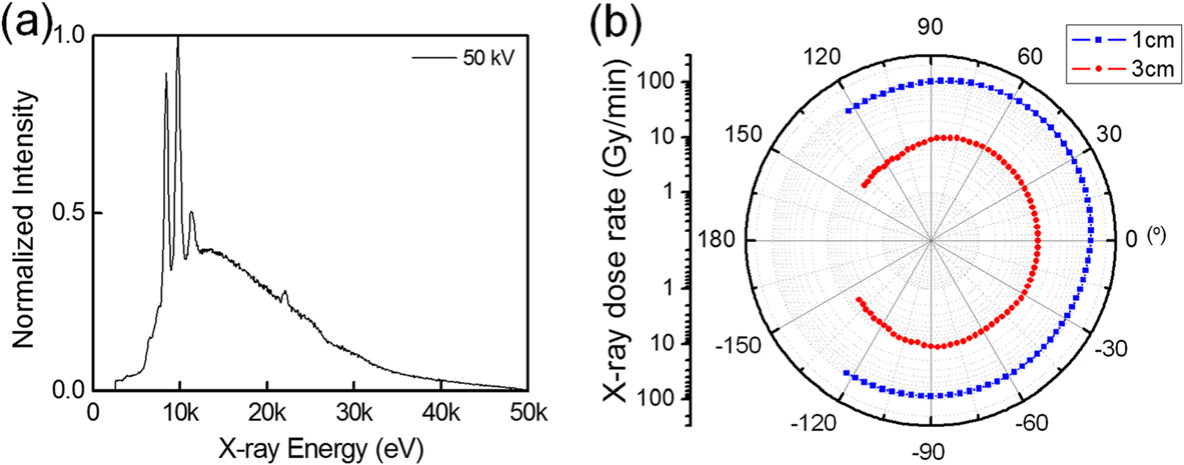
**X-ray spectrum and dose rate distributions of the miniature X-ray tube. **(**a**) X-ray spectrum of the miniature X-ray tube. The spectrums are normalized by the highest peak signal of 9.7 keV W L_β1_ characteristic X-ray. (**b**) X-ray dose rate distributions of the miniature X-ray tube measured at 1 cm (blue filled square) and 3 cm (red filled circle) in air.

Figure 3a displays the energy spectrum of the X-rays generated from the miniature X-ray tube operating at 50 kV. The spectrum was measured with an X-ray spectrometer (Amptek XR-100 T-CdTe, Amptek Inc., Bedford, MA, USA). The spectrum includes broad *bremsstrahlung* X-rays with energies of up to 50 keV and a few characteristic X-rays at 8.4, 9.7, and 11.3 keV that respectively correspond to L_α1_, L_β1_, and L_γ1_ of the W target [21]. Figure 3b shows the spatial distribution of the X-ray dose rate measured at 1 cm apart from the X-ray tube in air. The X-ray dose rate decreases slightly from 108.1 Gy·min^−1^ at an angle of 0° to 102.8 Gy·min^−1^ at ±60° and to 86.1 Gy·min^−1^ at ±120°. The intensity difference over the range of 240° was *ca*. 20%. This indicates that X-rays are comparatively uniformly produced in space, which is useful for the applications of brachytherapy or intracavity radiation imaging such as intraoral diagnosis.

**Figure 4:**
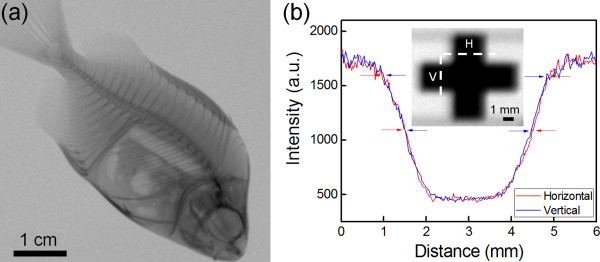
**XR images of a small raw fish and copperplate and intensity profiles. **(**a**) XR image of a small raw fish acquired at a 60-kV tube voltage. (**b**) XR image of a 0.1-mm thick copper plate with a magnification factor of 1.25. (**c**) Intensity profiles along the vertical and horizontal lines in the XR image of the copper plate.

Figure 4a shows the XR image of a small raw fish taken using the miniature X-ray tube and a CMOS photodiode array detector (Vatech Xmaru0505CF, Humanray Co., Ltd., Gyeonggi-do, South Korea; pixel pitch 24 μm) with the magnification factor of 1.02 (distance between the X-ray tube and the small raw fish is 15.0 cm). The tube voltage was 60 kV, and the X-ray exposure time was 0.1 s. As can be seen in the XR image, the bone structures and the internal organs, as well as the outline of the fish, are clearly observed. Since high enough X-ray intensity is produced from the miniature X-ray tube, such a clear XR image could be achieved in a short time.

To further evaluate the performance of the miniature X-ray tube, the X-ray focal spot size was measured following the European standard EN 12543–5. An XR image of a copperplate (thickness 0.1 mm) was taken with a magnification factor of 1.25 (Figure 4b). The image profiles in both the horizontal and vertical directions were analyzed, and from the analysis, the X-ray focal spot size was calculated to be 3.72 mm in the horizontal direction and 3.64 mm in the vertical direction. The focal spot size of the X-ray corresponds to the electron beam size at the X-ray target. Therefore, the measurement results for the X-ray focal spot size suggest that the inner diameter (7 mm) of the present miniature X-ray tube can be reduced, and accordingly, the X-ray tube can be further minimized.

## Conclusions

In summary, we have demonstrated a vacuum-sealed miniature X-ray tube based on CNT field-emission electron source. The X-ray tube can be operated up to 70 kV, and high-dose X-rays are generated with a comparatively good spatial dose distribution. Due to the small electron beam size in the X-ray tube, the prototype X-ray tube can be further miniaturized. We believe that such a vacuum-sealed miniature X-ray tube can be used for various industrial and medical diagnostic/therapy purposes.

## Competing interests

The authors declare that they have no competing interests.

## Authors' contributions

SHH carried out the design and fabrication of the experimental setups and drafted the manuscript. HJK participated equally in the fabrication of the miniature X-ray tube, in the data acquisition of the field emission and the X-ray dose, and in the preparation of the manuscript. JMH participated in the preparation of the carbon nanotube field emitter and assisted in the experiments. SOC supervised the whole study. All authors read and approved the final manuscript.

## Authors' information

SHH has a Ph.D. in Nuclear Engineering and is a president of a ventured company. HJK and JMH are MS students. SOC is a professor of Nuclear Engineering.
